# Synergistic effect of ultrasound and antimicrobial solutions of cecropin P1 in the deactivation of *Escherichia coli* O157:H7 using a cylindrical ultrasonic system

**DOI:** 10.1038/s41598-023-37198-7

**Published:** 2023-07-07

**Authors:** Maya Fitriyanti, Saeed Bagherzadeh, Ganesan Narsimhan

**Affiliations:** 1 School of Life Sciences and Technology, Institut Teknologi Bandung, Bandung, 40132 Indonesia; 2 Biosciences and Biotechnology Research Center, Institut Teknologi Bandung, Bandung, 40132 Indonesia; 3 School of Materials Engineering, Purdue University, West Lafayette, IN 47907 USA; 4 Department of Agricultural and Biological Engineering, Purdue University, West Lafayette, IN 47907 USA

**Keywords:** Biotechnology, Microbiology

## Abstract

This study investigates the synergistic effect of ultrasonication and antimicrobial action of antimicrobial peptide cecropin P1 on the inactivation of *Escherichia coli* O157:H7 in a cylindrical ultrasonication system. The inactivation of *E. coli* at pH 7.4 was performed using: ultrasonication (14, 22, and 47 kHz), cecropin P1 (20 µg/mL), and a combination of both. We found the treatment at 22 kHz, 8W for 15 min of exposure and a combination of ultrasound at higher frequency (47 kHz, 8 W) and cecropin P1 for one minute of exposure were more efficient, reducing the cell density by six orders of magnitude, compared to individual treatments (ultrasound or cecropin P1 only). Dye leakage studies and transmission electron microscopy further validated these results. A continuous flow system was designed to demonstrate synergism of ultrasonication with antimicrobial peptide Cecropin P1 in the inactivation of *E. coli*; synergism was shown to be more at higher ultrasonication frequencies and power levels. Acoustic cavitation by ultrasonic treatment could drastically improve microbial deactivation by antimicrobial peptides cecropin P1 by increasing their ability for pore formation in cell membranes. A continuous ultrasonication and antimicrobial peptides system can lead to an energy-efficient and economical sterilization system for food safety applications.

## Introduction

Thermal processing is still considered a superior method in the food industry to preserve food and extend shelf life; however, it is an energy-intensive process that results in a loss of food quality. To overcome this limitation, various novel alternative methods have been developed to replace at least partially thermal processing to minimize the loss in food quality at adequate safety levels. Ultrasound has been a part of actively emerging technologies in food processing. The physical and biological effect of sound waves generated from high-frequency ultrasound (100–300 kHz) has been investigated for years, as discussed in an early study by Loomis and colleagues^1,2^. However, high-frequency ultrasound (more than 100 kHz) has not been used extensively for food preservation and processing except for food quality monitoring and diagnostic purposes^3–5^. Depending on its operating parameters (frequency, energy intensity, exposure time) and food types, ultrasound may have a wide-ranging impact on food components and food sensory qualities, both positive and critical results^6–10^. Sound waves with lower frequency (20–100 kHz) yet of higher energy, which is referred to as “conventional power ultrasound,” has been shown to destroy microbial cells with minimum adverse effects on food quality such as vitamins, taste, and color^8–12^.

The primary antimicrobial effect of ultrasound is due to intense acoustic cavitation generated from the sound wave. Ultrasound alone can make some bacteria inactive, but it requires high power to achieve complete deactivation. For preservation purposes, combining with other physical or chemical treatments can lower the processing cost and enhance its effectiveness. To minimize the thermal effect on food, ultrasound-assisted with temperature (thermosonication), pressure (manosonication), or a combination of both (manothermosonication) has been proven to be effective in reducing microbial levels compared to thermal or ultrasound preservation alone^13,14^. Various lab-scale studies using milk, fruit, and vegetable juices have shown that ultrasound-assisted technologies have the advantage of inactivating more bacterial cells with a minimum adverse effect on food sensory characteristics compared to conventional heat treatments^15–22^. On the therapeutic application, ultrasound's most researched antimicrobial effect is the co-application with conventional antibiotics. Several investigations demonstrate that a combination of low-intensity and low-frequency ultrasound and antibiotics is more effective in the deactivation of bacteria than antibiotics alone^23–25^.

Antimicrobial peptides (AMPs) are naturally found in various organisms as part of innate immunity. The unique characteristics of AMPs are their small size (15–40 amino acids) and charge (often overall positive). They also disrupt cell membranes^26^. AMPs have raised broad research interest due to their ability to combat antibiotic resistance and the potential for the replacement of antibiotics^26,27^. For food application, it is essential to use naturally derived AMPs that do not exhibit cytotoxicity. Based on animal cell culture studies, cecropin P1 is known to have no cytotoxicity^28^. Antimicrobial peptides at low concentrations kill bacteria by pore formation in the cell membranes. Thus, transient pores formed by ultrasound cavitation should enhance antimicrobial activity. Our previous investigation has shown that a combination of longitudinal ultrasound or probe type (frequency 22 kHz) and AMP melittin in phosphate buffer media is more efficient in reducing cell density (CFU/mL) of *Listeria monocytogenes* up to four order magnitude compared to melittin or ultrasound alone^29^. A combination of longitudinal ultrasound (22 kHz) and another classic AMP cecropin P1 was able to reduce cell density (CFU/mL) of *E. coli* O157:H7 up to five orders of magnitude in orange juice and milk^16^.

Following our previous study, the primary motivation of this research is to investigate the synergistic effect of a cylindrical ultrasound and a classic AMP cecropin P1 on antimicrobial activity against *E. coli* O157:H7. The *E. coli* O157:H7 is the most commonly identified Shiga toxin-producing *E. coli* (STEC) that can cause severe foodborne disease. Generally, the inactivation of microorganisms by ultrasound depends on many factors, including frequency, ultrasonic power and wave amplitude, temperature, sample volume, composition and physical properties of food, type ultrasound, and microorganism characteristics^8,10,30,31^. For example, the frequency influences the formation and size of cavitation bubbles. At higher frequencies, the acoustic cycle is shorter, giving less time for cavitation bubble formation; therefore, more bubbles with smaller sizes are generated and collapse with less energy^32–34^.

In this study, a cylindrical ultrasonic processing unit was used to demonstrate the effect of ultrasound frequency and intensity on the synergistic effect of AMP. The design of this cylindrical system is based on the work of Borthwick et al.^35^. The ultrasonic processing system for cell disruption that is commercially available is the probe-based transducer with an attached sonotrode with an activated resonance mode of 20–22 kHz. The design of an ultrasonic transducer having a lower or higher resonance frequency than the standard 20–22 kHz could be challenging. The cylindrical ultrasonic transducer is less bulky than the probe system and would be convenient for handling smaller sample volumes and for use outside the laboratory. Also, there is an inherent risk of hazardous foam formation due to the immersion of the probe in a liquid sample containing pathogens^35^. The cylindrical ultrasonic device used in this study is also capable of continuous processing that can treat larger sample volumes. Therefore, this study aimed to investigate the performance of the cylindrical ultrasonic device against *E. coli* in combination with antimicrobial peptides for more efficient cell disruption and potential food preservation applications.

## Materials and methods

### Materials

Cecropin P1 (3.338, 86 g/mol) was purchased from Sigma-Aldrich (Saint Louis, MO) as a lyophilized powder with 95% purity. Brain Heart Infusion (BHI) medium was purchased from Neogen (Lansing, MI). The *E. coli* O157:H7 was obtained from Microbiology Laboratory, Department of Food Science, Purdue University, and calcein were purchased from Sigma Aldrich (Saint Louis, MO). In addition, 1,2-Dimyristoyl-sn-glycerol-3-phosphorylcholine (DMPC), Cholesterol, and hexadecyl hydrogen phosphate (DHP) with 99% purity were purchased from Avanti Polar Lipids (Alabaster, AL). The Piezoelectric cylinders with specified resonance frequencies of 14 kHz, 22 kHz, and 47 kHz were purchased from Steiner & Martins Inc. (Doral, FL) to make the cylindrical ultrasonic transducer.

### Methods

#### *E. coli* preparation

Preparation of *E. coli* O157:H7 was described elsewhere^16^. Briefly, *E. coli* O157:H7 was grown in BHI (Neogen, MI) media until it reached 10^9^ colony-forming units (CFU) per mL at 37 °C as measured by the plate count method.

#### Design of cylindrical ultrasonic system

**Figure 1:**
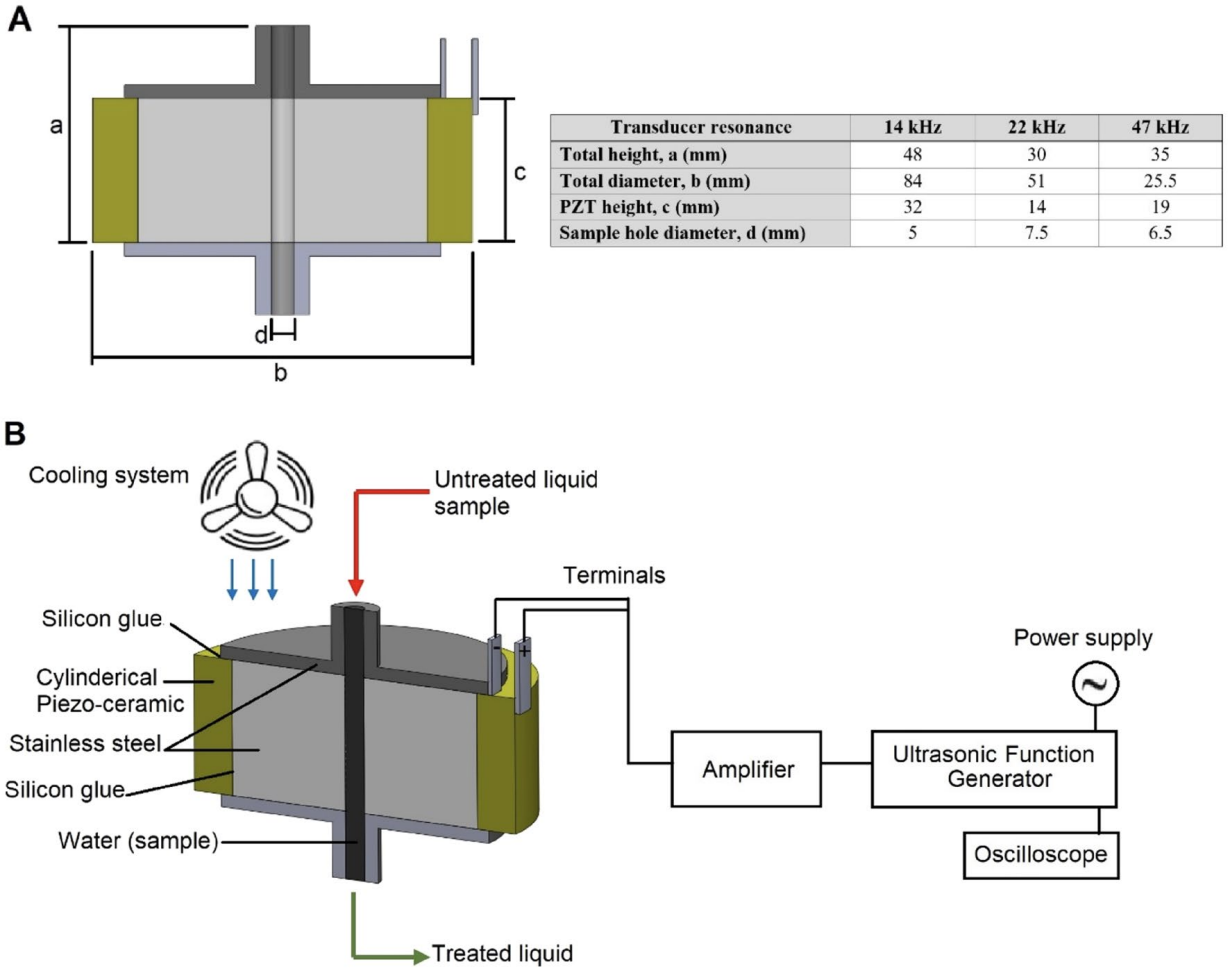
(**A**) The sectional view of a cylindrical ultrasonic transducer with the indicated dimensions, (**B**) The schematic view of continuous liquid treatment using the ultrasonic processing method.

Three different cylindrical ultrasonic transducers (14, 22, and 47 kHz) for ultrasonic processing systems have been designed and built based on Borthwick et al.^35^. Figure 1A illustrates a plan view with the system dimensions parameters. Figure 1B shows the schematic view for the continuous flow ultrasonic processing system. The ultrasonic transducer was driven by an amplifier (RF amplifier model 150A100B, AR, Souderton, PA) and a function generator (Agilent model 33120A, Keysight Technologies, Santa Rosa, CA), which provided a sinusoidal signal. An oscilloscope is used to verify the working frequency (OWON SDS5032E, Zhangzhou, China). Cooling fans was fitted around the transducer to cool the system. Voltage amplitude was measured using a multimeter (Fluke, Everett, WA) with constant gain input and frequency to determine the ultrasound power level.

#### Experimental procedure and analysis

##### Ultrasound experiment

For the batch system, inactivation of *E. coli* O157:H7 in PBS media was conducted using three different treatments: Ultrasound only for 5, 10, and 15 min, cecropin P1 only (20 µg/mL), and a combination of ultrasound with cecropin P1. The concentration of cecropin P1 20 µg/mL was chosen based on its minimum inhibitory concentration^36^ . The geometry and size of the sample container is a cylinder with dimension as mentioned in Fig. 1. The volume of cell suspension is 2 mL for all frequencies (14, 22, and 47 kHz). External fans are used to control the temperature during sonication. The treatment is done semicontinuous with 1 min on and 1 min off during the total treatment time. The initial cell density before all treatments is 10^9^ CFU/mL. The treated *E. coli* samples were then grown on a BHI agar plate at 37 °C for 24 h to determine the number of viable cells per mL (CFU/mL) described previously^16,29^. For the continuous flow system (22 kHz), residence time was maintained at 6, 10, 15, and 34 min, and the number of viable cells was determined using the same method.

##### Transmission electron microscopy

Morphological changes in the bacterial cell after treatment with different ultrasonication frequencies (14, 22, and 47 kHz) were analyzed using transmission electron microscopy (TEM) as described previously^16,29^.

##### Liposome preparation

The liposome is employed as a bacterial mimic in dye leakage experiments due to cecropin P1 and ultrasonication experiments. The procedure for preparing liposomes consisting of DMPC, cholesterol, and DHP in a molar ratio of 5:4:1 encapsulated with calcein dye is described elsewhere^37,38^. The vesicle suspension was forced through a polycarbonate filter to form uniform lamellar liposomes and measured by Zeta sizer (Malvern Instrument, Worcestershire, UK).

##### Fluorescence dye leakage

The fluorescence of calcein dye released from liposome subjected to ultrasound and cecropin P1 treatments was measured based on the previously described protocol^38^. Liposome loaded with calcein dye was treated with ultrasonication and cecropin P1 for 5, 10, and 15 min. The sample was then transferred to Spectrofluorometer (Flexstation II, Molecular Device, USA), and the fluorescence intensity was subsequently measured. The fluorescence intensity of calcein was normalized by the maximum intensity obtained by releasing all calcein from the liposome by adding Triton X-100^38,39^. Fluorescence intensity of leaked calcein is due to pore formation in a liposome due to different treatments and therefore is a measure of deactivation of bacterial mimic.

#### Statistical analysis

Analysis of variance (ANOVA) with Tukey’s test was carried out to determine any significant differences (*p* < 0.005) among the treatments. The ultrasound and dye leakage experiments were conducted in triplicates, and the mean values with standard deviations (SD) were recorded.

## Results and discussion

### Effect of ultrasound power

**Figure 2:**
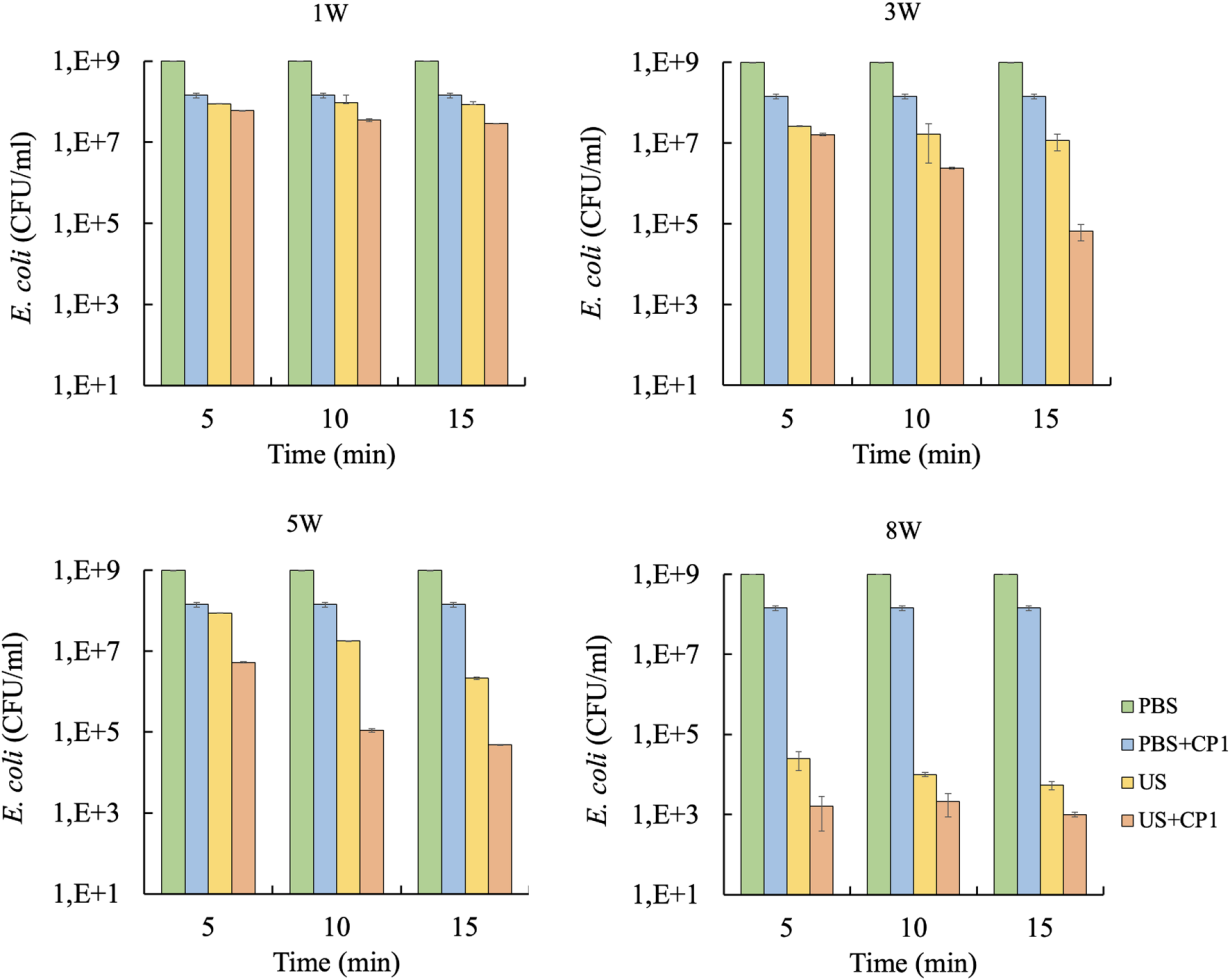
Effect of ultrasound power levels on synergistic effect against *E. coli* at a fixed frequency (22 kHz) using a batch system. PBS: *E. coli* without any treatment, PBS + CP1: *E. coli* treated with cecropin P1 only, US: *E. coli* treated with ultrasound only, US + CP1: *E. coli* treated with a combination of ultrasound and cecropin P1. Power density: 1 W (0.5 W/mL); 3 W (1.5 W/mL); 5 W (2,5 W/mL); 8 W (4 W/mL). Tukey’s test: PBS vs PBS + CP1: not significant; PBS vs US: significant difference (** *p* < 0.01); PBS vs US + CP1: significant difference (** *p* < 0.01); PBS + CP1 vs US: significant difference (* *p* < 0.05); PBS + CP1 vs US + CP1: significant difference (** *p* < 0.01); US vs US + CP1: not significant.

Figure 2 shows the effect of different power levels on the inactivation of *E. coli* cells using ultrasound, cecropin P1, and a combination of both at a fixed frequency of 22 kHz. As expected, more deactivations occur for a longer treatment time, although the difference is less significant at 10 and 15 min. Based on ANOVA statistical analysis, the effect of different power levels (1 W, 3 W, 5 W, and 8 W) and treatment types (ultrasound, cecropin P1, and a combination of both) showed significant difference (*p* < 0.05) in the deactivation of *E. coli*. For example, in ultrasonic treatment at a power level of 5W, the cell density decreases from an initial value of 10^9^ CFU/mL to 8.8 × 10^7^ CFU/mL, 1.8 × 10^7^ CFU/mL, and 2.2 × 10^6^ CFU/mL for treatment times of 5, 10, and 15 min, respectively, as shown in Fig. 2. In addition, more cells are deactivated as the power level increases. Under ultrasound treatment for 15 min, cell density decreases to 8.6 × 10^7^ CFU/mL from the initial value of 10^9^ CFU/mL for a power level of 1W. In contrast, the cell density for a power level of 5W is much lower at a value of 2.2 × 10^6^ CFU/mL. As expected, for treatment with cecropin P1 only (in the absence of ultrasound), more deactivation occurred at longer treatment times, resulting in decreased cell density. It is interesting to note that cell density was lower for combined treatment compared to either ultrasound-only or cecropin P1-only treatments. For example, for a power level of 5W and 10 min treatment time, cell density was 1.1 × 10^5^ CFU/mL for combined treatment compared to 1.8 × 10^7^ CFU/mL for ultrasound and 1.4 × 10^8^ CFU/mL for cecropin P1. This result indicated synergism between ultrasonication and cecropin P1 treatments. However, the synergism was more pronounced for intermediate power levels of 3W and 5W (*p* < 0.05), especially at longer treatment times. For power levels of 1W and 3W, there was negligible temperature rise for all treatment times. For higher power levels (5W and 8W), the temperature rise was significant (from 23 to 35 °C) only for 15 min treatment time.

Ultrasonication leads to pressure waves of ultrasonication frequency in the liquid medium. The amplitude of these waves is higher at higher power inputs^32^. At sufficiently high-power intensities, ultrasonication has been known to destroy microorganisms and enzymes in food and break down microstructures^32–34^. Power level is one of the critical factors affecting the efficiency of ultrasound treatment. Low pressure caused by these pressure waves results in the formation of bubbles in the medium due to cavitation. These bubbles collapse, thus resulting in shock waves that emanate radially into the surrounding medium. They interact with neighboring bacterial cell membranes and push the phospholipid heads apart, forming transient pores. High power produces higher pressure amplitude, thereby causing a more significant number of bubbles due to cavitation and more violent collapse.

In the previous study^16^, we presented the result from cell deactivation using a commercial probe-type ultrasonic system, which is used to sonicate a larger sample volume of 5 mL compared to the cylindrical system that has a smaller sample volume of 2 mL. If we compare the result from our previous study at a fixed frequency of 22 kHz and power density of 40W/5 mL and 8W/2 mL, respectively, for the probe and cylindrical system, and with an initial cell density of 10^9^ CFU/mL, the cylindrical system was able to deactivate cell faster (15 min compared to 30 min) and one order of magnitude higher at lower power level. A similar result has been observed by Borthwick et al.^35^. Their result showed that a tubular ultrasonic processing device (267 kHz, 36W) has six times faster protein release and higher cell deactivation per 10^7^ *Saccharomyces cerevisiae* yeast suspension compared to a 20 kHz probe system within the 60 s. This observation might be due to the radial mode of vibration inward in the cylindrical system, which concentrated pressure at the center of the cylinder. The advantage of a cylindrical ultrasonic processing system also made it possible to sonicate a smaller sample without foaming, which is hard to avoid in a conventional 20 kHz probe-type device.

### Effect of ultrasound frequencies on synergistic effect

Based on results in Fig. 2, the synergistic effect is more pronounced at a power level of 3W and higher for a longer exposure time (15 min) (*p* < 0.05). The most significant synergistic effect can be seen at a power level of 5W (*p* < 0.05). As we described previously, the ultrasonication method kills bacterial cells by forming transient pores in the cell membranes due to shock waves generated by the collapse of bubbles formed by cavitation. Thus, transient pores formed by ultrasonication should result in the diffusion of intracellular matter and enhancement of antimicrobial activity^29^. Pore formation due to ultrasonication can also be observed later in TEM images presented in Fig. 6. In vegetative forms, Gram-negative bacterial cells such as *E. coli* are more susceptible to ultrasound treatment compared to Gram-positive because they have thinner peptidoglycan^40,41^.

**Figure 3:**
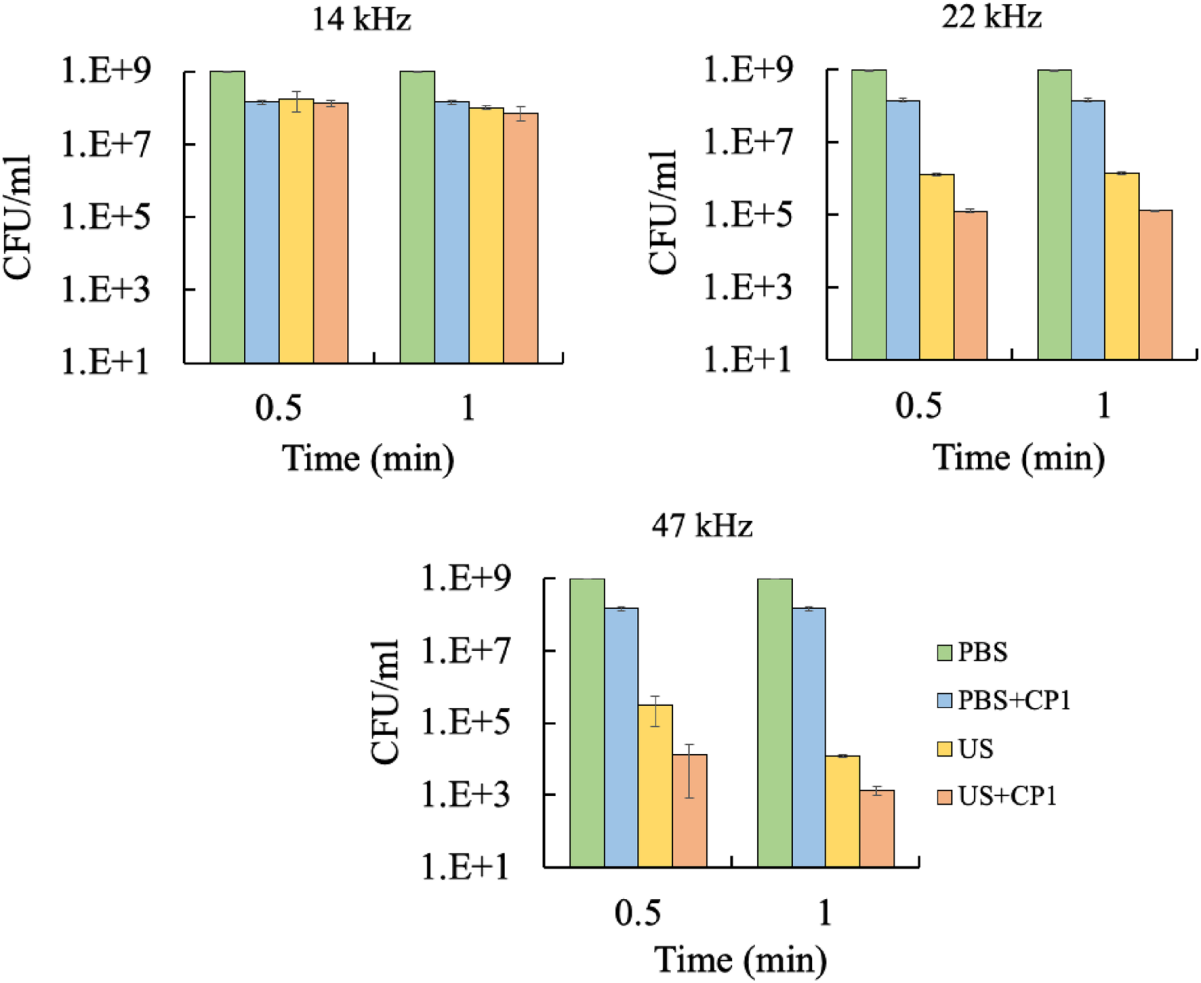
Effect of ultrasound frequencies on synergistic effect at a fixed power level of 8W (4W/mL) using a batch system. Blue: *E. coli* without any treatment, orange: *E. coli* treated with cecropin P1 only, grey: *E. coli* treated with ultrasound only, yellow: *E. coli* treated with a combination of ultrasound and cecropin P1. Tukey’s test: PBS vs PBS + CP1: not significant; PBS vs US: significant difference (** *p* < 0.01); PBS vs US + CP1: significant difference (** *p* < 0.01); PBS + CP1 vs US: not significant; PBS + CP1 vs US + CP1: significant difference (* *p* < 0.05); US vs US + CP1: not significant.

Figure 3 shows the effect of frequencies at a fixed power level of 8W on the deactivation of *E. coli* using ultrasound, cecropin P1, and a combination of both. It must be noted that treatment times of only 0.5 and 1 min were investigated and were, therefore, much shorter than those for experiments reported in Fig. 2. Comparison of deactivation at different frequencies could only be carried out for short treatment times (up to 1 min) since longer treatment times at a higher frequency of 47 kHz resulted in excessive heating of the sample. Deactivation is more at higher frequencies. For example, for an ultrasound treatment time of 1 min, the cell density values were 1.0 × 10^8^, 1.4 × 10^6^, and 1.3 × 10^4^ CFU/mL at 14, 22, and 47 kHz, respectively. Cell deactivation was significantly pronounced at higher frequencies (*p* < 0.05) than at 14 kHz at these short treatment times. Combined ultrasound and cecropin P1 treatments were more effective than individual treatments (*p* < 0.05). For example, for 0.5 min treatment at 47 kHz, cell density for combined treatment was 1.3 × 10^4^ CFU/mL compared to 3.1 × 10^5^ CFU/mL for ultrasound only and 1.4 × 10^8^ CFU/mL for cecropin P1 only. Also, synergism was highest for the highest frequency of 47 kHz, especially at 0.5 min treatment time. Results indicate that cell deactivation for a concise treatment time of 1 min at a higher frequency of 47 kHz is comparable to deactivation at a lower frequency of 22 kHz for a much longer treatment time of 15 min. This result suggests that ultrasonication efficiency increases dramatically at higher frequencies.

The formation of transient pores by sonication facilitates cell death by reducing the energy barrier for the formation and growth of pores by cecropin P1^38^. At sufficiently high intensities, these transient pores can be large enough to cause considerable leakage of intracellular matter, thus leading to cell death. At lower intensities, however, cecropin P1 will adsorb onto the inner lining of the transient pore, with the hydrophilic side chains lining the inside of the pore and the hydrophobic side chains pointing towards the lipid tails. Further adsorption of cecropin P1 onto preexisting pores would result in the growth of these pores, eventually leading to leakage of intracellular matter and cell death^42,43^. Hence the synergistic effect between ultrasonication and antimicrobial peptide action. The synergistic effect was observed for longitudinal probe treatment and radial ultrasonic processing using a cylindrical probe. A study done by Ozuna et al.^44^ also reported the synergistic effects of antimicrobial peptides of thurincin H (40 μg/mL) and probe-type power ultrasound (frequency of 20–25 kHz, the nominal power of 150 W) in milk and orange juice which effectively inactivate *L. innocua* and *E. coli*, with higher levels of inactivation than those observed when applying these technologies separately.

The frequency of sound waves influences the number and size of cavitation bubbles. The acoustic cycle at higher frequencies is shorter, giving less time for cavitation bubble formation and producing smaller cavitation bubbles than at lower frequencies. Also, increasing the ultrasonic frequency while maintaining the same ultrasonic power will result in a more significant number of smaller cavitation bubbles. As a result, at higher ultrasonic frequencies, more bubbles formed with a smaller size which collapsed with less energy^15,32^. Figure 3 demonstrates that at 1 min of exposure time, the cell deactivation is higher at a higher frequency (47 kHz). The synergistic effect between ultrasonication and cecropin P1 was still visible at this frequency. It could deactivate cells up to six orders of magnitude comparable to a 22 kHz case within 15 min of treatment. However, at 14 kHz, the deactivation is less pronounced compared to 22 and 47 kHz. This result suggests that the increased bubble concentration at a higher frequency is the predominant effect on deactivation. Previous studies have also shown that higher frequencies than 20–25 kHz were able to deactivate more bacterial and algal cells^45,46^. The extent of sonication time is limited at 47 kHz due to temperature build-up during cavitation. Therefore, the experiment is carried out for less than 2 min. A better cooling strategy is needed to overcome this.

### Continuous flow system

**Figure 4:**
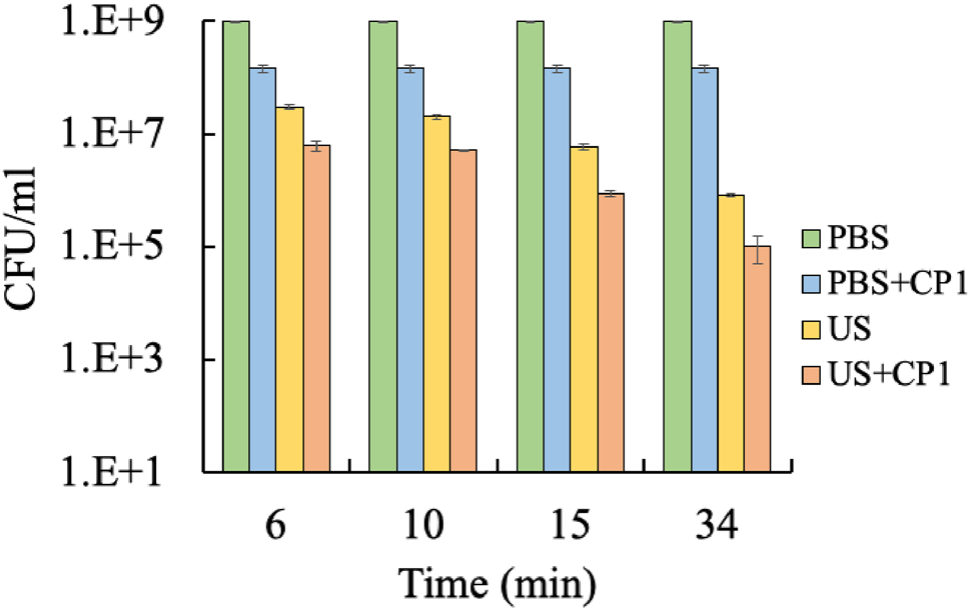
Effect of residence time on synergistic effect at a fixed frequency (22 kHz) and fixed power level (8W) using a continuous ultrasonic processing system. PBS: untreated *E. coli*, PBS + CP1: *E. coli* treated with CP1 only, US: *E. coli* treated with ultrasonication only, US + CP1: E. coli treated with ultrasonication and CP1. Tukey’s test: PBS vs PBS + CP1: not significant; PBS vs US: significant difference (** *p* < 0.01); PBS vs US + CP1: significant difference (** *p* < 0.01); PBS + CP1 vs US: significant difference (* *p* < 0.05); PBS + CP1 vs US + CP1: significant difference (** *p* < 0.01); US vs US + CP1: not significant.

Deactivation experiments were performed in a continuous flow ultrasonic processing system using a 22 kHz cylindrical transducer at different residence times (6, 10, 15, and 34 min). The viable cell count after treatment in a continuous flow system is presented in Fig. 4. Higher residence time (34 min) is more effective in deactivating more *E. coli* cells (up to four orders of magnitude). For example, for ultrasound treatment only, cell density decreased from 3.0 × 10^7^ CFU/mL to 8.1 × 10^5^ CFU/mL when the residence time was increased from 6 to 34 min (Fig. 4). The continuous system also observed synergism between ultrasound and cecropin P1 treatments. Synergism was more pronounced at residence times of 15 and 34 min. For example, combined treatment at 6 min residence time reduced cell density to 6.2 × 10^6^ CFU/mL compared to 3.0 × 10^7^ and 1.4 × 10^8^ CFU/mL for ultrasound and cecropin P1, respectively. On the other hand, at a residence time of 34 min, combined treatment gave 9.9 × 10^4^ CFU/mL compared to 8.1 × 10^5^ and 1.4 × 10^8^ CFU/mL for ultrasound and cecropin P1, respectively. However, the continuous system is less effective in deactivation, as evidenced by cell density reduction only by four orders (10^9^–10^5^) as opposed to a decrease by six orders (10^9^–10^3^) for 15 min batch treatments.

The synergistic effect and cell deactivation are less in a continuous flow system (Fig. 4) if we compare this result with the batch system (Figs. 2 and 3) at a comparable power level of 8W. This observation might be due to the lower actual pressure field inside the bacterial suspension. The fixtures in a continuous system might act as an anchor that damped the transducer's vibration. The advantage of using a continuous system over a batch system is the flexibility to treat larger sample volumes without temperature build-up.

### Dye leakage due to treatment with cecropin P1 and cylindrical ultrasonication

**Figure 5:**
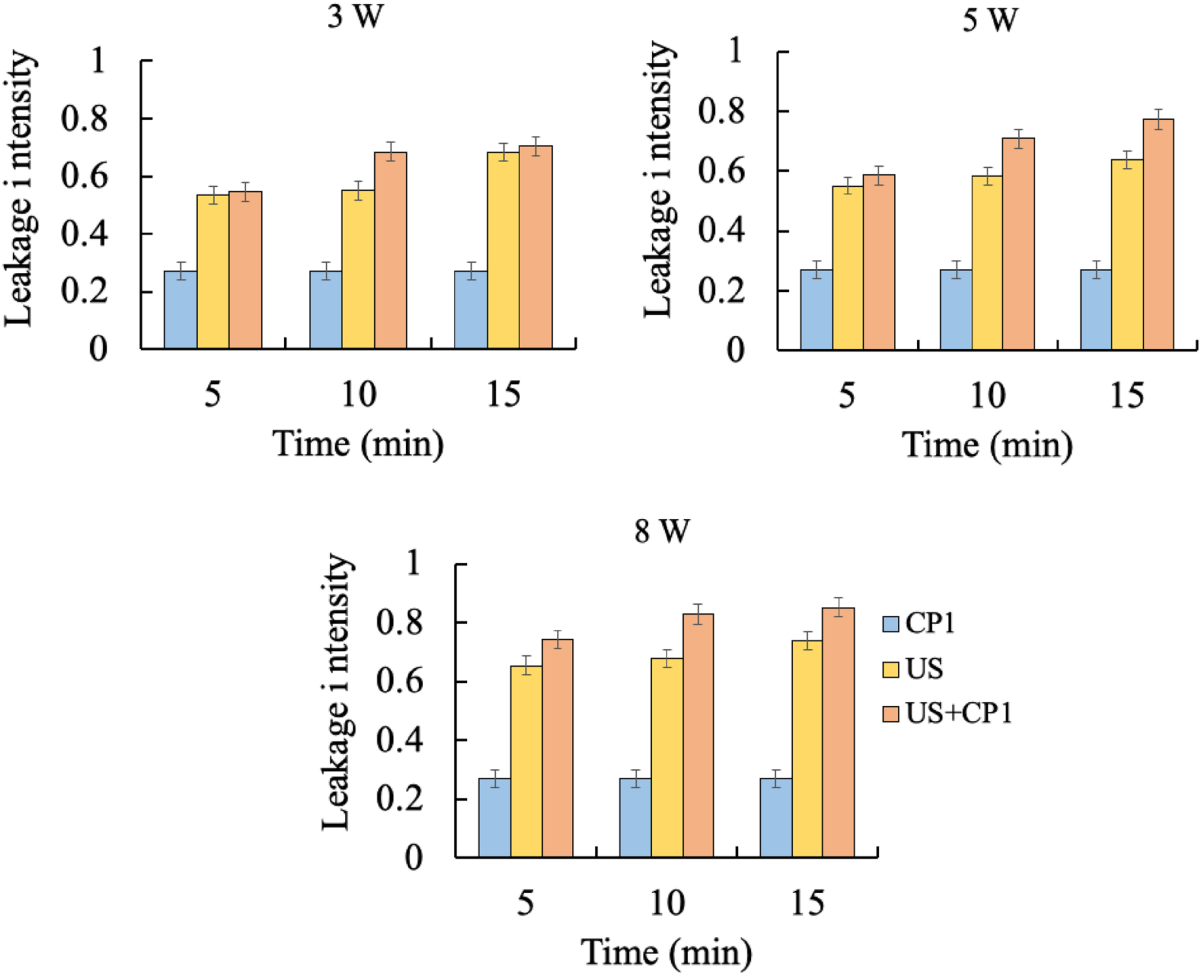
Maximum calcein leakage after treatment with cecropin P1 (20 µg/mL) and ultrasonication (3, 5, and 8W). The DMPC/cholesterol liposome was loaded with calcein dye and treated with cecropin P1, ultrasonication, or a combination of both for 5, 10, and 15 min, and then measured the dye leakage intensity. CP1: *E. coli* treated with CP1 only, US: *E. coli* treated with ultrasonication only, US + CP1: *E. coli* treated with ultrasonication and CP1. Tukey’s test: CP1 vs US: significant difference (** p < 0.01); CP1 vs US + CP1: significant difference (** *p* < 0.01); US vs US + CP1: significant difference (* *p* < 0.05).

As explained in the methods, the fluorescence intensity of leaked calcein from liposome was measured at different times from the moment the sample was transferred to Spectrofluorometer. The fluorescence intensity of the sample was higher than the background when it was transferred to Spectrofluoremeter, thus indicating that dye leakage had occurred due to different treatments. The calcein dye continued to leak, with the fluorescence intensity increasing slowly with time, eventually reaching a constant value at a sufficiently long time (10 min). The final steady-state fluorescence intensity is a measure of total dye leakage and is therefore compared for different treatments in Fig. 5. Fluorescence intensity increased with ultrasound treatment time, thus indicating more damage to liposomes (more leakage), consistent with cell deactivation results reported earlier. The results also showed that dye leakage is higher for liposomes treated with a combination of cecropin P1 and ultrasonication, demonstrating synergism between the two.

Calcein leakage intensity from liposomes depends on acoustic pressure and exposure time, although the differences are minor (Fig. 5). The maximum leakage intensity of this marker dye is slightly higher after treatment with a combination of ultrasound and cecropin P1 at 8W for 15 min compared to 3W and 5W, which is also consistent with the cell viability reduction. As explained previously, transient pore formation due to sonication will cause this dye to leak into the environment and finally approach equilibrium. Similar results were also observed in some studies which correlated ultrasound-induced bioeffect with energy density^47,48^.

### Effect of ultrasonication on morphology of bacterial cell

**Figure 6:**
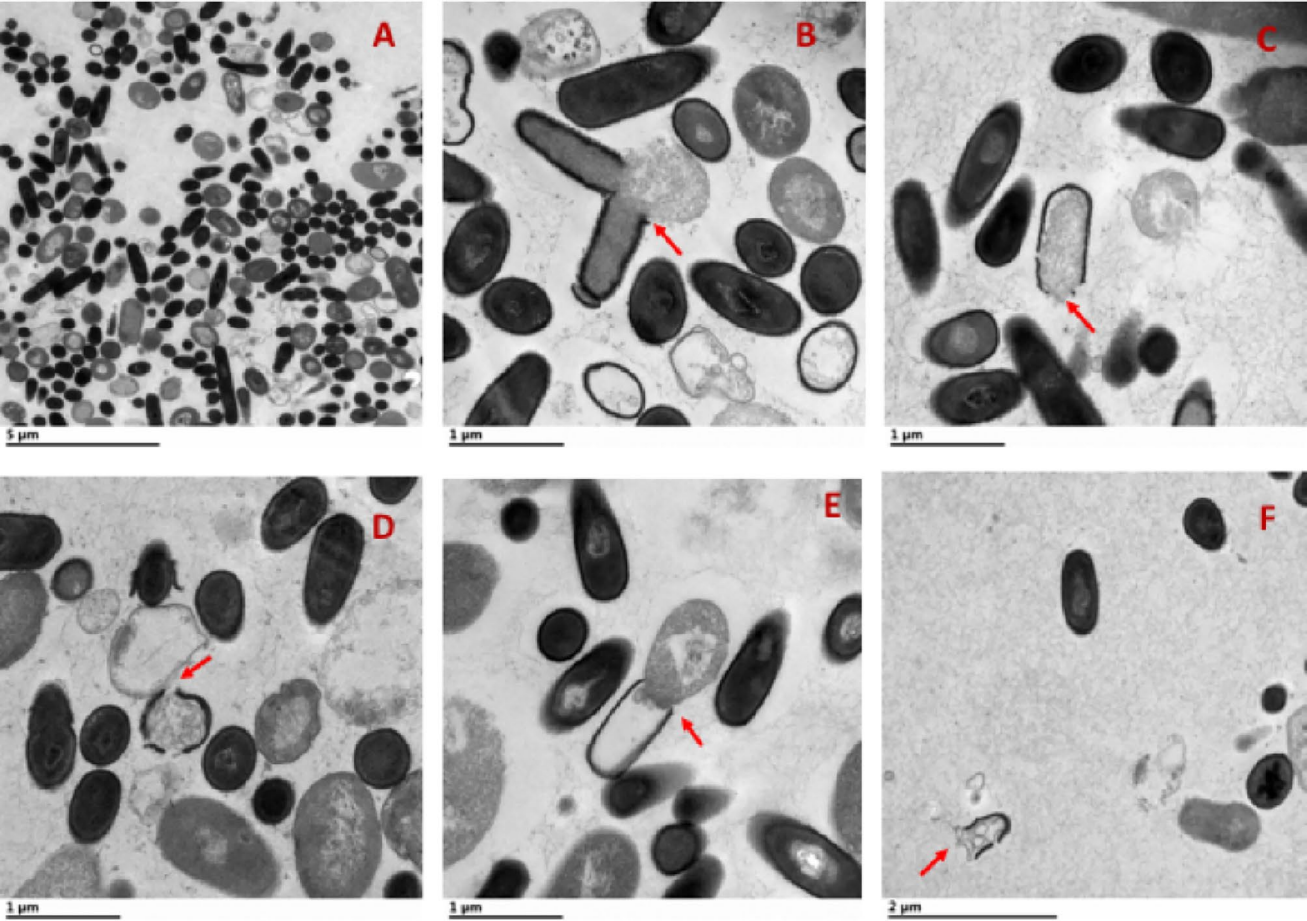
TEM show single or multiple pore formations (red arrow) on *E. coli* due to ultrasound and cecropin P1 treatment. (**A**) control, (**B**) 14 kHz, (**C**) 22 kHz, (**D**) 47 kHz, E, and F: a combination of ultrasound (22 kHz) and cecropin P1.

Morphological changes in *E. coli* cells occurred after exposure to ultrasonication at different frequencies (14, 22, and 47 kHz) and a combination of ultrasound and cecropin P1, as can be seen from TEM images in Fig. 6. The cell wall is disrupted, and the cytoplasmic material is released to the extracellular medium when exposed to ultrasonication and combined treatment. The pore formation, which resulted in leakage of intracellular material, was observed (pointed by red arrow) when *E. coli* cells were exposed to these treatments. In Fig. 6C, D and F, we can see multiple pore formations due to 22, 47 kHz, and a combination of ultrasound (22 kHz) and cecropin P1, respectively.

Gram-negative bacteria such as *E. coli* have a thinner cell wall. Thus, it is more sensitive to ultrasound treatment. Based on our previous study^16^, cecropin P1 alone could not completely deactivate *E. coli* at a minimum inhibitory concentration and a higher concentration, as indicated by the presence of some intact cells. The cylindrical system also observed a similar result (Fig. 6). Transmission electron study done by other investigators also showed disintegration of Gram-negative bacteria *E. coli B* and *Klebsiella pneumonia* due to probe ultrasound treatment (frequency of 20 and 40 kHz)^45^.

## Conclusion

The deactivation of *E. coli* in PBS (pH 7.4) was performed using three different treatments: (1) ultrasound (22 kHz) at different power levels (1, 3, 5, and 8 W) and different exposure times (5, 10, and 15 min), (2) cecropin P1 (20 µg/mL), and (3) combination of both. The number of deactivated cells (CFU/mL) increases as the power level increases, and a synergistic effect is observed at a power level of 3W and higher. A combination of ultrasound and cecropin P1 treatment at 8W for 15 min reduced most of the cells (up to six orders of magnitude reduction) compared to individual treatments. Our results on the effect of different frequencies (14, 22, and 47 kHz) also show that a combination of higher frequency (47 kHz) and cecropin P1 for one minute of exposure time was able to deactivate more cells (up to six orders of magnitude reduction) compared to combined treatment at 14 and 22 kHz.

A continuous flow ultrasonic processing system using a cylindrical transducer at 22 kHz with a power level of 8W demonstrated that longer residence time increases cell reduction. Cell reduction up to five orders of magnitude was achieved for the residence time of 34 min. The synergistic effect and cell deactivation at a comparable power level are less in the continuous flow system. This result might be due to a distribution of residence times experienced by the fluid in the cylinder.

The dye leakage experiment and TEM confirmed the synergistic effect of ultrasonication and cecropin P1. TEM images show a single and multiple pore formation due to ultrasound and cecropin P1 treatments which lead to cell death.

## Data Availability

All data and materials are the result of research and are available from the corresponding author upon reasonable request and are appropriately cited in the manuscript.
